# Smart Garment Fabrics to Enable Non-Contact Opto-Physiological Monitoring

**DOI:** 10.3390/bios8020033

**Published:** 2018-03-29

**Authors:** Dmitry Iakovlev, Sijung Hu, Harnani Hassan, Vincent Dwyer, Roya Ashayer-Soltani, Chris Hunt, Jinsong Shen

**Affiliations:** 1 Wolfson School of Mechanical, Electrical and Manufacturing Engineering, Loughborough University, Loughborough LE11 3TU, UK; D.Iakovlev@lboro.ac.uk (D.I.); H.Hassan@lboro.ac.uk (H.H.); V.M.Dwyer@lboro.ac.uk (V.D.); 2 National Physical Laboratory, Hampton Road, Teddington TW11 0LW, UK; ashayerroya@gmail.com (R.-A.S.); chris.hunt@pireta.co.uk (C.H.); 3 Pireta Limited, Hampton Road, Teddington TW11 0LW, UK; 4 Textile Engineering and Materials Research Group, School of Design, De Montfort University, Leicester LE1 9BH, UK; jshen@dmu.ac.uk

**Keywords:** imaging photoplethysmography (iPPG), smart garment fabric, light emitting diode (LED), heart rate measurement, signal processing, motion artefacts

## Abstract

Imaging photoplethysmography (iPPG) is an emerging technology used to assess microcirculation and cardiovascular signs by collecting backscattered light from illuminated tissue using optical imaging sensors. The aim of this study was to study how effective smart garment fabrics could be capturing physiological signs in a non-contact mode. The present work demonstrates a feasible approach of, instead of using conventional high-power illumination sources, integrating a grid of surface-mounted light emitting diodes (LEDs) into cotton fabric to spotlight the region of interest (ROI). The green and the red LEDs (525 and 660 nm) placed on a small cotton substrate were used to locally illuminate palm skin in a dual-wavelength iPPG setup, where the backscattered light is transmitted to a remote image sensor through the garment fabric. The results show that the illuminations from both wavelength LEDs can be used to extract heart rate (HR) reaching an accuracy of 90% compared to a contact PPG probe. Stretching the fabric over the skin surface alters the morphology of iPPG signals, demonstrating a significantly higher pulsatile amplitude in both channels of green and red illuminations. The skin compression by the fabric could be potentially utilised to enhance the penetration of illumination into cutaneous microvascular beds. The outcome could lead a new avenue of non-contact opto-physiological monitoring and assessment with functional garment fabrics.

## 1. Introduction

Increasing demand for remote physiological measurement in areas such as healthcare, emergency response services, elite sports, and recreational fitness has stimulated research into numerous “smart” and wearable devices. A usual approach is to integrate physiological sensors, e.g., electrocardiograms (ECG) and photoplethysmographs (PPG), into all-in-one smart-watches or other body-worn hardware, which are attractive to the rehabilitation, sports, and fitness markets [1]. However, another simple and cost-effective remote monitoring method is attaching a small sensor and power circuitry to smart garments, while all sensing, processing, and data distribution nodes are set remotely around the patient. This approach eliminates the need to secure hardware to the patient’s body to prevent involuntary tampering with the sensor, which in turn leads to unobtrusive patient monitoring in hospital wards or homes and to higher user satisfaction.

Photoplethysmography (PPG) has been widely adopted and used as an inexpensive technique to acquire vital physiological signs including heart rate, respiration cycles and blood oxygen saturation (SpO2). A camera-based method, also known as imaging (iPPG) or remote (rPPG) photoplethysmography, has been used to demonstrate the possibility of remote pulse rate extraction, where tissue surface is illuminated by ambient [2] or artificial light [3], and modulated backscattered light is captured by an image sensor, e.g., a digital camera. The significant downside of iPPG, compared to the traditional contact PPG, is the need of a powerful light source to spotlight the region of interest (ROI) [4]. The quality of an iPPG signal is also susceptible to the directionality and uniformity of such illumination [5]. However, the greatest limitation of the traditional iPPG setup is its limited ability to sense physiological signs from ROIs obstructed mostly by clothes. This drawback is also associated with the fact that the incident light is being absorbed, reflected, or highly attenuated by tightly knitted or woven fabric, making the collection of modulated backscattered light virtually impossible.

**Figure 1:**
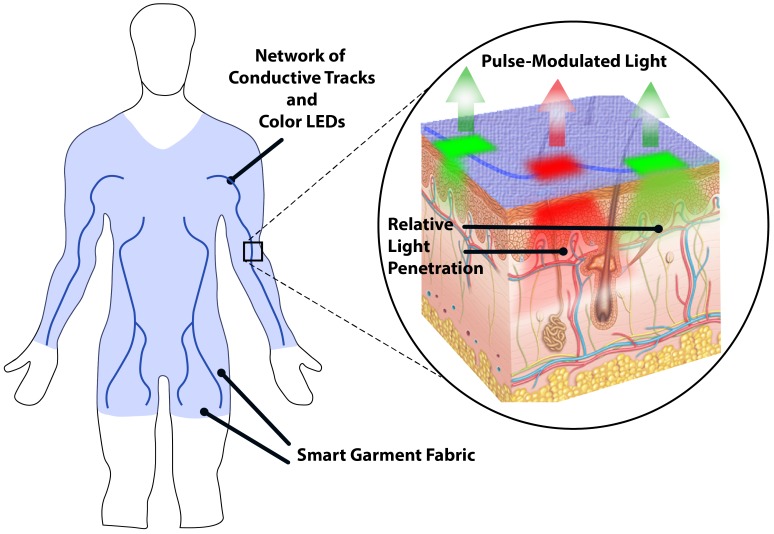
Illustration of the experimental smart garment with conductive tracks and light emitting diodes (LEDs) facing the tissue surface. Generated illumination penetrates skin tissue and becomes modulated by pulsatile blood flow in the microvasculature. Backscattered light is then captured and analysed by the remote optical system (not shown).

An alternative approach is to employ the latest development in “smart” garment fabrics integrated with a grid of miniature light emitting diodes (LEDs) facing the tissue surface. This grid would create a uniformly illuminated zone around ROIs while allowing the modulated backscattered light to escape through the textile fabric and reach a remote image sensor (Figure 1). The goal of this research work was to study how effective smart garment fabrics could be in capturing physiological signs in a non-contact mode. To reach this aim, the objectives were to (a) assess the feasibility of inlaying a piece of garment with LED modules for local tissue illumination, (b) analyse the performance of a dual-wavelength iPPG setup by evaluating the backscattered light transmitted to an image sensor through the garment fabric, and (c) estimate basic physiological signs (heart rate) and compare it with a reference contact PPG probe.

## 2. Materials and Methods

### 2.1. Smart Garment Fabrics and Hardware Setup

The research model consists of two sets: (a) a dual-wavelength light source placed on the garment (prepared by the National Physical Laboratory, London, UK) and (b) a remote camera sensor and processing electronics established by the Photonics Engineering Group, Loughborough University, Loughborough, UK.

Surface-mount LEDs (TRL-2D15, Truelight Co., Taiwan) with a 0.6 × 0.3 mm footprint with peak wavelengths of 525 and 660 nm with a typical half-power bandwidth of 15 nm were selected due to their high efficacy/size ratio. Their wide-angle radiation pattern of ∼120° provided greater illumination coverage over a larger area using fewer LED chips. The spectral properties were quantified using a calibrated spectrometer (USB4000, Ocean Optics, Dunedun, FL, USA) and an optical power meter (Model 835, Newport, RI, USA), which were synchronised with the light source to record parameters during video acquisition. This approach allows monitoring of illumination source spectral behaviour over time and under various LED forward currents for improved experiment control and repeatability.

**Figure 2:**
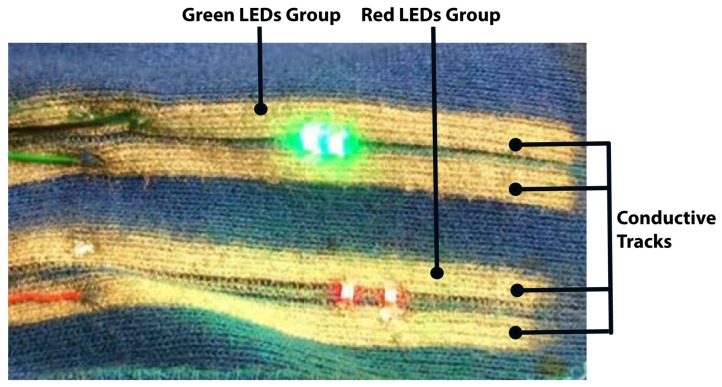
An example of the experimental smart garment fabric with conductive tracks and LEDs.

The conducive tracks were then printed on the fabric, and the parallel paths to power each group of LEDs were spaced by 0.4 mm to avoid short circuits, while the groups were separated by a 5 mm gap. The LEDs as illumination sources were lastly attached to the fabric (Figure 2). Compared to utilising traditional metal wires to power the light emitters, the proposed solution allowed the material to be flexible and stretch without significant stress cracks.

**Figure 3:**
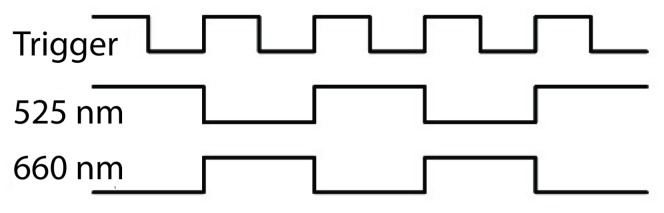
Relative timings of the control unit (CU) triggering the camera and LEDs.

The number and relative position of LEDs in each group varied depending on the required optical output and skin tissue area, but generally, LEDs of the same wavelength were spaced in-line by 2–3 mm. A custom constant-current LED driver was designed in-house to power LEDs with the ability to vary forward current in the 2–40 mA range with <2% distortion in order to achieve different levels of light intensity. A control unit (CU) based on the SAM3X8E microcontroller (Atmel, San Jose, CA, USA) alternately powered each wavelength group of LEDs in a time-division multiplexing (TDM) scheme, producing 10 ms light pulses and triggering the start of exposure on the imaging sensor (Figure 3).

**Figure 4:**
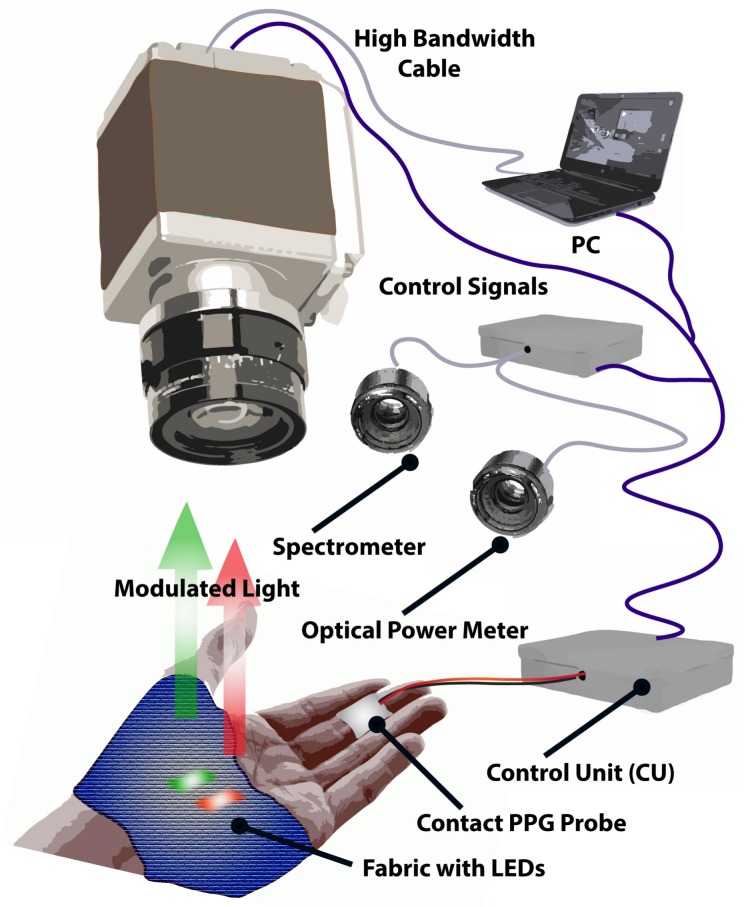
Hardware setup for data acquisition. The control module (CU) is responsible for alternately activating each LED group and triggering camera frame exposure synchronously using control signals. The intensity of each LED group can be tuned via the CU. The spectrometer and optical power meter are also synchronised by the CU to monitor LED optical output stability over time and between consecutive runs. Parameters are set and monitored on a PC using a custom graphical interface.

A non-contact imaging sensor comprised of a sCMOS monochrome camera (Orca Flash V2, Hamamatsu Co., Hamamatsu, Japan) set to a 16 bit dynamic range with an effective resolution of 2048 × 2048 pixel (Figure 4). A set of prime optical lenses with a focus distance of 50 , 85, and 100 mm (Planar T ZF-IR, Zeiss, Jena, Germany) with an extended spectrum range (400–950 nm) were selected to provide several image magnification options and achieve higher optical resolution. The camera was set from a personal computer workstation via a Camera Link interface board using HCImage software (Hamamatsu Photonics, Hamamatsu, Japan). Once triggered by the CU, the camera read out a predefined number of images, which were then transferred to a PC via a Camera Link frame grabber (Firebird, Active Silicon, Iver UK). Each frame was exposed for 20 ms, resulting in an overall frame rate of 50 or 25 fps per wavelength group.

A contact PPG probe (cPPG) based on an integrated optical sensor (SFH 7050, OSRAM, Munich, Germany) was also placed in the proximity of the smart garment fabric, but outside of the camera’s field of view to avoid optical disturbance. The cPPG probe was driven by the CU at 100 Hz, while the digitised signal was transferred and stored on a PC via a USB interface.

### 2.2. Fabric Preparation

**Figure 5:**
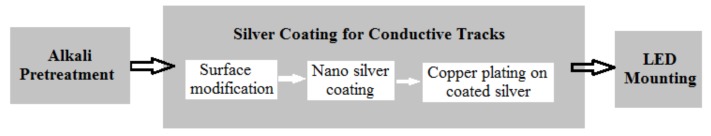
Fabric preparation stages.

The knitted cotton fabric with a dry weight of 147.4 g/m ${}^{2}$ was supplied by Cotton Incorporated, USA. The knitted cotton fabrics were scoured, bleached, and dyed with reactive dye. Silver-coated conductive tracks were produced by using *in situ* reduction of silver nitrate using a controlled inkjet printing method. Four tracks designed by Loughborough University Photonics Engineering Research Group were deposited on the fabric using the procedure shown in Figure 5.

- *Alkali Pretreatment:* Cotton fabric was pretreated with a 10 wt % aqueous NaOH solution at room temperature for 20 min followed by rinsing with a copious amount of distilled water. All samples were dried prior to the experiments.
- *Surface Modification using PDADMAC:* A 1 wt % aqueous solution of PDADMAC (Polydiallyldimethylammonium chloride) was used to modify the cotton surface. After thoroughly wetting the fabric with the solution, it was dried at 60 °C in an oven for 5 min in order to evaporate any remaining water molecules.
- *Nano Silver Synthesis on the Cotton Fabric:* A silver solution of 0.025 M silver nitrate (99.99% AgNO ${}_{3}$) was prepared. Next, the cotton fabric was wetted with 0.1 mL of a 1.61 × 10${}^{-4}$ M NaBH ${}_{4}$ solution per 1.5 g of fabric. The silver nitrate solution was then added to the fabric (typically 10 μL/64 mm^2^). Each track was covered with silver nanoparticles after three consecutive reductions.
- *Copper Electroless Plating:* Copper electroless plating was achieved using a Circuposit 3350-1 electroless copper bath (Chestech Ltd., Rugby, UK) at a pH value of 12. The immersion duration was 25 min at a temperature of 46 °C. The copper thickness was considered to be 1.25 μm. Immersion silver was then used to protect the copper tracks from oxidation.
- *LED mounting:* All LEDs were mounted on the fabric using conductive adhesive. LEDs were then covered with a WBP conformal coating to protect them from external damage and excessive wear during testing.

### 2.3. Measurement Site Selection

The reliability and quality of pulse oximetry rely on a careful detection of blood pulsations from capillary or peripheral vessels while minimising signal contribution from venous blood and other stationary or non-modulating mediums, such as bones and muscle tissues. A human palm was selected as a potential measurement site due to its high concentration of blood vessel per unit area, as well as the relative ease to incorporate an illumination source into a casual cloth accessory, e.g., gloves. The challenge in designing such a smart garment was to position the illumination source above the skin area with underlying arterial vessels to maximise the chances of detecting blood pulsations from capillary or peripheral vessels. If, on the contrary, the illumination source was placed in an area with low or no concentration of capillary or peripheral vessels, the detected backscattered light would contain lower or almost no variation of fractional blood volume, leading to poorer pulsatile waveforms (namely a PPG signal) and a lower signal–noise ratio (SNR).

According to previous research work [6,7], the achievable penetration depth for incident light, which should be backscattered to the photodetector, does not exceed 3 mm for the skin tissue in the spectral range of 450–950 nm. An experiment with NIR illumination source concluded that even though such a long wavelength can theoretically penetrate more than 10 mm *in vivo* in the tissue, the obtained pulsatile curve was attenuated to 50% by only 1.05 mm of bovine muscle tissue [8]. Therefore, the prerequisites for obtaining a reliable PPG signal with a higher SNR and experiment repeatability should lead to (a) identification of a strong pulsatile arterial vessel in proximity to the superficial layer in the region of interest, and (b) relative transparency of the epidermis and dermis layers to the applied illumination wavelength.

**Figure 6:**
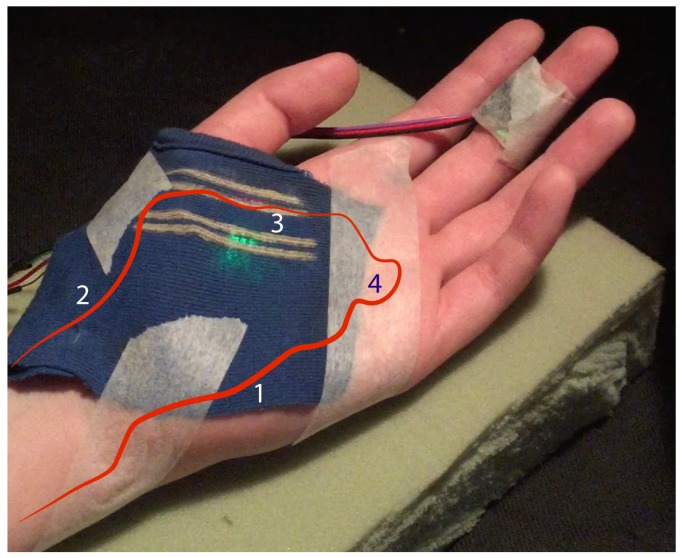
A representation of arterial vessel network of an inner adult palm. (1) Ulnar artery. (2) Radial artery. (3) Small superficial palmar branch of the radial artery. (4) Superficial palmar branch. Experimental fabric with two wavelength LEDs secured on a subject’s palm. All LEDs are facing downwards the skin surface. A contact photoplethysmography (PPG) sensor secured on the middle finger was used for validation of imaging PPG (iPPG).

The small superficial palmar branch of the radial artery (Label 3 in Figure 6) initially demonstrated the theoretical potential for better PPG signals and more reliable detection of arterial blood pulsations due to its proximity to the tissue surface. However, a closer examination of the area revealed underlying subcutaneous muscle tissues, so a PPG sensor located close to this area was predicted to be susceptible to motion artefact noise, generated by altering an optical path length, and ballistocardiographic (BCG) artefacts associated with cardiac-related micro-motion of the skin surface [5]. The hypothesis set in this experiment was that a promising observation site would be a large flat area above the superficial palmar branch (Label 4 in Figure 6) in the center of a palm. In the present setup, areas around the thumb and in the center of the palm were investigated.

### 2.4. Experimental Protocol

The experimental smart garment fabric was evaluated on 10 subjects (aged 21–45, 4 females and 6 males) at the Photonics Engineering Research Group, Loughborough University, UK. These subjects belonged to the Group II and III of the skin classification system developed by Fitzpatrick [9]. No health hazards were identified with the usage of cotton fabric, printed conductive tracks, or LEDs on the skin surface. The experiment exclusion criterion was set to the subjects with pacemakers, due to potentially uncontrolled and untested influence of electronic equipment in the laboratory. The experiment protocol was approved by the Ethics Committee at Loughborough University, UK, and all subjects signed a consent form prior to the experiment.

Each subject was asked to rest his/her right palm on a support with the palm facing up. A piece of the experimental garment fabric was placed on the skin surface and secured by medical adhesive tape (3M, UK). A contact PPG (cPPG) probe was also strapped to the middle finger to act as a reference signal. The camera and lens assembly was positioned above the palm on the adjustable arm in the range of 0.45–1.1 m above the skin surface to provide levels of image zoom. The lens was focused and centered on the area of the highest LED illumination level, verified by the real-time histogram on HCImage software. The spectrometer and optical power meter were also positioned above the palm and focused on the area around the LED groups. The experiment took place in a dark optical laboratory to avoid any ambient light interference, aside from the smart garment LEDs, exposing the camera sensor. The ambient temperature was controlled at 25 ± 2 °C. The image frames were captured during 4 separate sequences, each lasting for 30 s at the rate of 50 frames per second (fps) or 25 fps per each LED group channel.

### 2.5. Signal Processing

**Figure 7:**
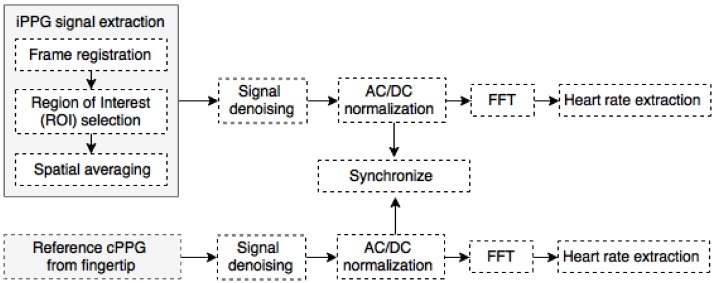
Signal processing pipeline for the remote iPPG and contact PPG (cPPG) signals.

All images captured by the camera and signals from the cPPG sensor were processed offline in MATLAB (MathWorks Inc., Natick, MA, USA) (Figure 7). The captured images were initially separated into two groups organised by the two channels of green and red LEDs used to illuminate the skin. Each frame was registered with respect to the first frame in the data set sequence using a cross-correlation method discussed in [10], which allowed for the stabilisation of still images and the dramatically reduction of motion artefacts. All individual pixels within a predefined region of interest (ROI) were averaged, providing a single aggregated pixel value per frame per LED wavelength. These signals would be referred to as $iPPG_{g}$ and $iPPG_{r}$ for 525 nm green and 660 nm red channels, respectively.

The reference signal from the contact probe (cPPG) was denoised by passing it through a Butterworth low-pass filter (7th order, cut-off frequency at 7 Hz). The relative separation of the cPPG probe from the smart garment fabric was 4.5–9 cm depending on the size of the subject’s palm, resulting in a small phase delay between cPPG and $iPPG_{g-r}$ signals. This delay was eliminated by manually offsetting the cPPG signal, so cardiac peaks and troughs for each cycle were in-phase with $iPPG_{g-r}$ signals, which were verified by peak detection and phase shift estimation.

All PPG signals were normalised by so-called AC/DC normalisation to provide invariance to LED brightness variations, achieved by firstly obtaining a low-frequency quasi-DC component ($PPG_{DC}$) via utilisation of a Butterworth low-pass filter (5-th order, cut-off at 0.5 Hz). Normalised signals $PPG_{AC/DC}$ were calculated as $PPG_{AC/DC}=\left(PPG_{raw}-PPG_{DC}\right)/PPG_{DC}$.

Heart rate (HR) was extracted in the frequency domain by obtaining a fast Fourier transform (FFT) of the normalised PPG signals. Firstly, cPPG and $iPPG_{g-r}$ signals were divided into sliding segments of 1000 and 250 samples, respectively, with a 5-sample shift, corresponding to 10 s. This was done to minimise the effect of heart rate variations over time, which could result in a wider fundamental pulse peak as seen in the FFT spectrum. Secondly, the position of the frequency component with the highest amplitude was located, which corresponded to the heart rate with the conversion factor of 1 Hz = 60 BPM. cPPG was assumed to be less susceptive to motion artefacts and less noisy due to its firm contact with the fingertip, so it was selected as a ground truth signal for the current experiment. Hence, one of the evaluation criteria included a correlation factor between HR derived from cPPG and $iPPG_{g-r}$ in the range 0–1.

The second evaluation criterion included noise component analysis in the frequency domain, adopted from [11]. Ideally, a sinusoidal-like PPG signal would have a clearly profound peak with sharp roll-off around its fundamental pulse frequency, followed by few harmonic components. These frequency components would be attributed to a clean PPG signal. The SNR was calculated to act as a quality metric, where the signal component was derived from the spectral energy within ±0.1 Hz of the fundamental heart rate frequency, and the noise was the remaining spectral energy in the 0–7 Hz range.

## 3. Results

**Figure 8:**
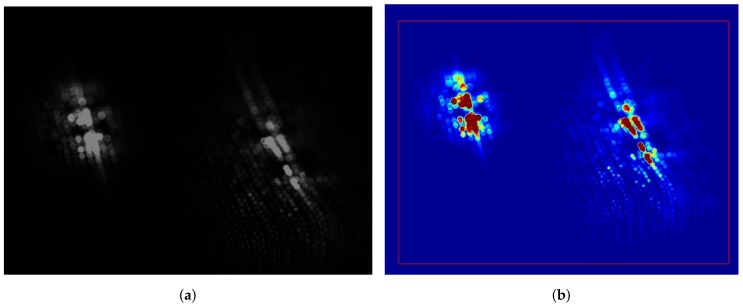
(**a**) A monochrome still image of a smart fabric sample (under magnification, 85 mm prime lens). Green 525 nm LEDs are on the left, red 660 nm LEDs are on the right. (**b**) A pseudo-colour transfer applied to a monochrome image to show regions of varying light intensity. The colours of red and blue represent the highest and lowest pixel values, respectively. The red rectangular frame is the selected region of interest (ROI).

Figure 8 shows the result of dual-wavelength iPPG with the experimental smart garment fabric. The green and red LED channels exposed the camera sensor independently one channel at a time, but their respective frames are stitched together in Figure 8a for the purpose of demonstrating their relative location. The pseudo-colour transfer was applied to the monochrome image to qualitatively assess backscattered light as shown in Figure 8b. The region associated with dark red was the area where LEDs were mounted on the smart garment fabric. Due to the transparent nature of the LED chip substrate, the direct high-intensity light travelled into the photodetector without interacting with the underlying tissue first. This resulted in “saturated” pixels, which did not contribute toward pulsatile signal since their intensity level was always at its maximum. The dark blue pseudo-colour was associated with regions that reflected no backscattered light or its level was comparable with the sensor’s instrumentation noise, so their contribution to pulsatile PPG signal was also zero. The areas with other pixel values were the regions of potential pulsatile backscattered light. The ratio of the saturated and dark pixels to pulsatile pixels was another parameter that determined the quality $iPPG_{g-r}$ signal.

### 3.1. Motion Artefacts

Applied cross-correlation image registration algorithm provided a significant reduction of motion artefacts when the palm was in a translational motion with respect to the camera sensor. The algorithm was unable to estimate the motion vectors correctly when the palm exhibited rotational motion, neither around its vertical axis nor during palm rolling from side to side. Side rolling had the strongest influence on the quality of the $iPPG_{g-r}$ signals, both in time and frequency domains. This observation was believed to be associated with alteration in the optical path when the palm and smart garment changed their orientation with respect to the camera lens.

The evaluation of motion robustness was not a primary objective for this study. Hence, the test protocol was re-designed to allow only the translation motion. This was achieved by modifying the palm support to avoid rolling. Rotational motion (around the vertical axis) did not degrade the iPPG signal quality due to the spatial averaging within the ROI. As can be seen in Figure 8, the black/blue regions contributed no pulsatile signal, so their location and orientation were not critical. Moreover, the backscattered light from a single wavelength light source was captured at any given time, so the relevant position of individual pixels made no difference, as long as the ratio of selected active to non-active (fully saturated or zero-valued) pixels remained fairly constant within the ROI from frame to frame. This situation, however, would not hold true in scenarios where spatial averaging over the entire frame region is not desirable, for example, iPPG imaging or blood perfusion mapping [12].

### 3.2. iPPG Signal Analysis

**Figure 9:**
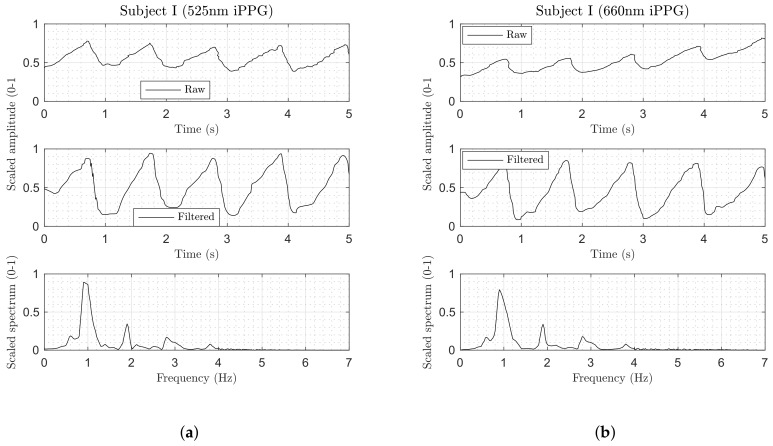

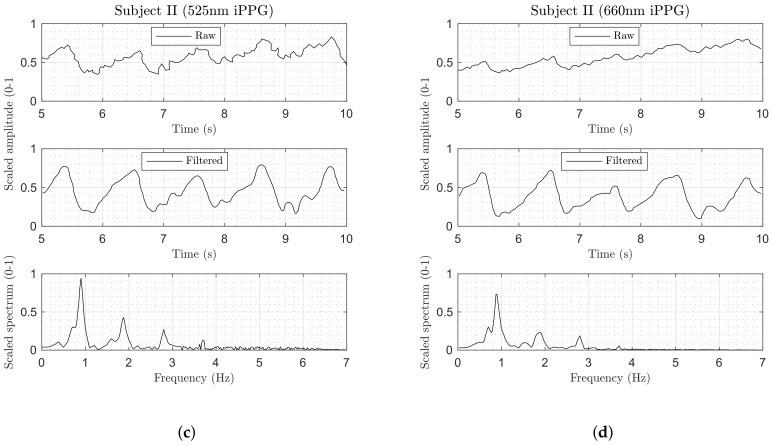
(**a**–**d**) Extracted physiological signals from an experimental dual-wavelength iPPG setup from two subjects. (top row) Spatially averaged and scaled signal. (middle row) Normalised, filtered, and scaled signal. (bottom row) Frequency content of filtered signal.

Figure 9 demonstrates a signal extracted from a 250-frame window (10 s at 25 fps) using the experimental smart garment fabric. Both channels of the dual-wavelength iPPG method demonstrated relatively similar morphology in the time domain, such as a triangular-like shape with a shallow ascent to the systolic peak and steep descent to the diastolic trough. $iPPG_{g}$ exhibited a higher peak-to-trough amplitude of the AC/DC-normalised signal compared to $iPPG_{r}$.

None of the observed iPPG signals contained a dicrotic notch (a small dip that follows the systolic peak), which was present and clearly visible in the reference cPPG. This lack could be attributed to a significantly slower sampling rate of the imaging sensor (25 Hz versus 100 Hz in cPPG), resulting in a decreased temporal resolution. When a dicrotic notch was delayed from the systolic peak by less than 40 ms (1/25 Hz) at an elevated heart rate (>85 BPM), it might not have been sampled by the sensor. The lack of the dicrotic notch in the $iPPG_{g-r}$ signal could also be explained by extremely heavy spatial averaging during signal processing. The individual pixels within the ROI could potentially preserve this notch, but the relevant phase and amplitude differences in the individual PPG signals across those pixels made the averaged signal appear “smoothed”, effectively masking the notch. The size of the kernel used to average pixels within the ROI directly influenced the iPPG signal morphology, which was confirmed by the observation made earlier in [4].

The frequency domain of $iPPG_{g-r}$ signals (bottom plot in Figure 9) demonstrated at least two well-defined harmonics of the fundamental heart rate frequency. Higher-order harmonics (3–4) in $iPPG_{r}$ attenuated more progressively compared to $iPPG_{g}$, where they decayed almost linearly. The majority of the spectral energy was concentrated in the 0–3 Hz range, with no high-frequency components beyond 4 Hz.

### 3.3. The Influence of LED Brightness Level on HR Calculation

**Figure 10:**
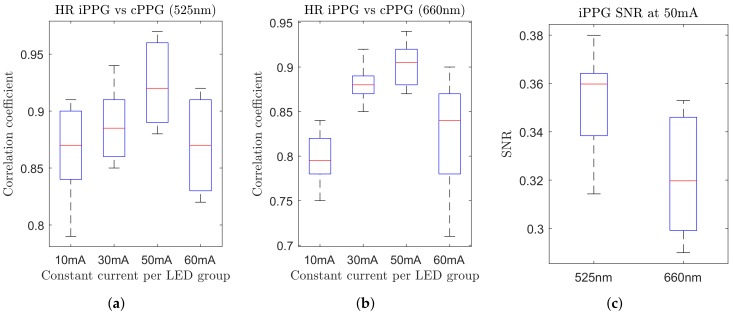
(**a**,**b**) Correlation coefficient of the calculated HR in iPPG and cPPG signals across 10 subjects under various LED current levels. (**c**) Anticipated SNR for both channels at 50 mA constant current. The signal is defined by the energy around a fundamental pulse-peak of ±0.1 Hz, and noise is the energy of the remaining spectrum.

In the conventional contact and remote iPPG, the invariance to the local illumination level variations is often achieved by means of AC/DC normalisation. The methodology is based on the fact that an increase in the incident light (from an illuminator to the skin surface) results in an enhanced pulsatile AC signal, as well an elevated DC level of the diffused backscattered light [13]. The experimental smart garment setup used in this study demonstrated a deviation from this assumption, where a shift in the forward current through each LED group resulted in a change in $iPPG_{g-r}$ signal quality and, therefore, the HR readings. Figure 10a,b demonstrate that at the low current level the correlation between the iPPG and reference cPPG HR readings was extremely low, reaching 0.85 and 0.8 for green and red iPPG channels, respectively, dipping to 0.76 for some subjects. The explanation for such a low correlation level ties with the fact that incident dim backlight illuminated a relatively small skin area, which might not contain a high concentration of superficial pulsatile blood vessels. Not all small sub-regions on the palm have the same potential for high-quality PPG extraction; this hypothesis was also confirmed in [11], where sub-regions with extremely low PPG SNR were excluded from further analysis. On the other hand, a high LED current resulted in brighter incident illumination and a larger proportion of sutured pixels within the ROI, leading to a poorer calculation of HR readings in the frequency domain.

The most promising current level was set at approximately 50 mA per LED group. This arrangement provided an optimal balance of pulsatile and non-pulsatile (zero-valued) pixels without optical saturation. The generated incident light covered an area of approximately 2.5–3 cm${}^{2}$, which was wide enough to include pulsating superficial capillaries. The full dynamic range of 16 bits per pixel was successfully used to detect micro-level changes in pixel values, resulting in a more detailed iPPG signal. Both green and red channels managed to provide high correlations (0.94 and 0.91, respectively) for the HR reading with respect to the reference contact cPPG. The correlation factor never fell short of 0.85, even for subjects with excessive motion artefacts.

SNR estimation of the $iPPG_{g-r}$ signals was used as another comparative mean. Figure 10c demonstrates a ratio of spectral energy around the fundamental pulse-peak to the remaining spectrum. The increased amount of in-band noise in the red $iPPG_{r}$ channel, which could not be suppressed by a simple passive filter, resulted in a lower level compared to the green channel. Since this project was only aimed at detecting HR, the observed separation of the fundamental pulse-rate frequency from the in-band was adequate for reliable HR estimation. However, for more complex computations, it would be advisable to employ an adaptive bandpass filter or derive a method that is immune to PPG signal distortions.

The experiments with higher current levels resulted in permanent LED degradation and eventually in failure due to uneven current sharing between individual LED modules. The experimental smart garment garments had all LEDs installed in parallel with no load resistors due to the overall complexity of printing conductive tracks and mounting small footprint components. Since LEDs are current-controlled devices, this arrangement resulted in some LEDs achieving more current and generating significantly more light output compared to other neighboring LEDs, leading to temporal instability in the optical output. It was evident that, in the event of one LED breaking into an open-circuit due to overheating, the rest of the LEDs in the same channel group would not be able to sustain increased load from the constant current driver, resulting in the entire chain being burnt out. Therefore, a modification to the LED connection arrangement was suggested for the next experiment stage.

### 3.4. Influence of Tissue Compression

In the first experiment, the smart garment fabric was positioned loosely on the subjects’ palm, exerting no pressure on the skin surface. An additional experiment was undertaken to evaluate the performance of the smart garment when it compresses the skin, for example, when integrated into gloves or other tight garments. The fabric was stretched to its maximum level and pressed against the palm, and secured in position by an adhesive medical tape. This resulted in the top surface being squashed and LEDs being pressed into the skin, which was confirmed in more pronounced tactile sensations reported by volunteers.

**Figure 11:**
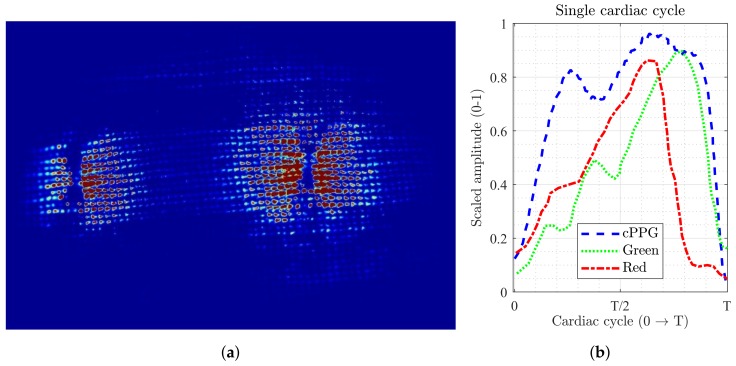
(**a**) Close-up look at the cotton fabric under tension. Backscattered light is allowed to escape through significantly wider slots between cotton threads. (**b**) iPPG signals appear less distorted and noisy, and the shape of a single cardiac cycle from both channels is comparable to the reference cPPG. Note that signals have been scaled individually to the 0–1 range.

The noticeable changes were clearly seen on the captured images (Figure 11a). Firstly, the distribution of the non-zero pixels changed from an oval-shaped (Figure 8b) to a more round-shaped cluster. The non-saturated pixels spread further away from the LEDs, providing more separation between pulse-modulated and saturated regions. Secondly, stretched garment fabric allowed significantly more backscattered light to pass between the cotton yarn, lowering the need for brighter LED illumination while still achieving a satisfactory iPPG signal quality.

The morphology of $iPPG_{g-r}$ signals demonstrated significantly higher pulsatile amplitude in both green and red channels (Figure 11b). The cardiac cycles were clearly defined, and the FFT-based heart rate estimation showed better correlation with the reference cPPG probe.

The downside of the fabric stretching was relatively low experiment repeatability. The fabric tension was restrained by the adhesive tape, and its orientation on the palm remarkably influenced the iPPG signal. The green channel showed the highest sensitivity to the applied skin compression in terms of iPPG quality, ranging from extremely strong to barely noticeable AC pulsations with a very insignificant change on the applied fabric pressure. Similar results were reported by pressing a transparent glass against the palm in the remote iPPG imaging under green light, where PPG signal strength and AC peak-to-trough amplitude both increased [14]. The red channel, on the other hand, was found to be less susceptible to variations in the superficial skin microvasculature structure, suggesting that it reached mostly undeformed deeper pulsatile blood vessels.

### 3.5. Influence of Tissue Site

The position of the fabric on the skin tissue played a crucial role in the quality of the detected $iPPG_{g-r}$ signals. Even with the same forward current and camera settings, a change in the fabric’s orientation resulted in a very explicit variation in iPPG quality. In contrast to the conventional remote iPPG where the backscattered light finds a direct optical path to the camera sensor, this experimental setup had a piece of fabric blocking backscattered light rays. Further examination of the fabric sample under a microscope revealed a heterogeneous structure of its knitted cotton threads with areas of tightly and loosely packed fibers. Those areas were observed to play the role of an optical filter, selectively blocking light depending on its relative orientation on the skin surface.

The experiments on finding an optimal location on the palm for the strongest iPPG signals were not conclusive. The hypothesis raised earlier with regard to potentially stronger signals around the superficial palmar branch was not confirmed fully. Due to a large number of influencing parameters (LED brightness, fabric tension and orientation, the individual structure of the microvascular bed, etc.), assessment of the superficial palmar branches of the ulnar and radial arteries showed no significant variability in the results.

### 3.6. Toward Clinical Validation

Simple principles of tissue optics have been utilised in clinical physiological monitoring for decades [15]. Starting with a contact transmission-mode probe, this technology has now been developed well enough to allow high-accuracy non-contact reflection-mode PPG signal acquisition [2,3]. However, to our knowledge, the feasibility of combining local skin-based light sources and a remote photodetector has not yet been proposed and evaluated. This, therefore, implies a new challenge to validate this method against widely accepted standard practices.

One of the proposed solutions is to position a clinically accredited reflection-mode contact PPG probe in close proximity to the smart garment sample. The active light source of such a probe should be optically isolated and shielded to prevent light rays from leaking and reaching the camera sensor, which would result in an optical disturbance. Moreover, the contact probe should be small enough not to interfere with natural body motion, but large enough to be attached to the skin surface by an adhesive medical tape, for example.

Local contact probe can be replaced by a medically approved fingertip pulse oximeter. Depending on the location of the smart garment sample, the local iPPG signal morphology and the one detected by a fingertip pulse oximeter could vary in signal quality due to the different concentration of blood vessels directly underneath the measurement cite, their relative depth, and the disruption in the optical path due to the presence of muscles, bones, or connective tissues. Additionally, the relative distance between the fingertip and the smart cotton cite has to be taken into account to solve for the phase delay associated with a propagating pulsatile wave.

Finally, ECG could be utilised to measure the wearer’s heart rate and compare it with the one obtained by the proposed method. Although the source of an ECG waveform is attributed to the changes in the heart’s electrical potential rather than blood volume changes, the average and instantaneous heart rates could be compared be synchronising both signals. Thorough validation against aforementioned standards is planned in a follow-up project.

## 4. Conclusions

This study demonstrated a new approach in the remote iPPG setup by incorporating the miniaturised LED modules into a piece of cloth, which had not been attempted and reported until now. Despite its simplicity, the design of such a smart garment requires careful optical and physiological design considerations. The investigation proved the possibility of remote heart rate readings with 87–92% accuracy of a comparable contact-based reference probe. The green 525 nm light, compared to the red 660 nm light, penetrates more shallowly into the microvascular bed, but provides a stronger and cleaner PPG signal. However, the morphology of green PPG signals is not preserved during direct pressure impact on the skin surface, making it more susceptible to external factors, such as garment extension or shrinkage.

This study also identified that a proportion of pulse-modulated backscattered light is retained by cotton yarn when the material is worn next to the skin. By stretching the material, the gap between neighboring threads can be increased, allowing more light to escape and reach the camera sensor. Pressing the fabric over the skin surface by stretching the fabric material also compresses tissue top surface, significantly improving the amplitude and AC/DC ratio of the extracted iPPG signal. The lack of the elastic and recovery properties of the 100% cotton fabric samples are thought to be causing experiment repeatability, but the use of an elastane-contained cotton fabric is suggested to improve these properties. A further investigation into compressive fabric is also advisable, as this approach could potentially provide higher-quality iPPG signals.

Lastly, the hardware modifications and redesign are required for LED conductive tracks. Light modules should be connected in-series where possible to achieve a constant brightness level across all LEDs in a single channel group. Further design considerations are recommended to sturdily secure LEDs to the fabric surface, making them less fragile and more sustainable to general wear and tear.

## References

[1] Patel S., Park H., Bonato P., Chan L., Rodgers M., Bonato P., Wallace J., Jeffers P., Taylor B., McSorley K. (2012). A review of wearable sensors and systems with application in rehabilitation. J. NeuroEng. Rehabil..

[2] Verkruysse W., Svaasand L.O., Nelson J.S. (2008). Remote plethysmographic imaging using ambient light. Opt. Express.

[3] Zheng J., Hu S., Azorin-Peris V., Echiadis A., Chouliaras V., Summers R. (2008). Remote simultaneous dual wavelength imaging photoplethysmography: A further step towards 3-D mapping of skin blood microcirculation. Proc. SPIE.

[4] Iakovlev D., Dwyer V., Hu S., Silberschmidt V. (2016). Noncontact blood perfusion mapping in clinical applications. Proc. SPIE.

[5] Moço A.V., Stuijk S., de Haan G. (2016). Motion robust PPG-imaging through color channel mapping. Biomed. Opt. Express.

[6] Huelsbusch M., Blazek V. (2002). Contactless mapping of rhythmical phenomena in tissue perfusion using PPGI. Med. Imaging.

[7] Tuchin V.V. (2015). Tissue Optics and Photonics : Light-Tissue Interaction. J. Biomed. Photonics Eng..

[8] Niklas M., Moser U., Buehrer A., Valentin R., Abicht J., Baschnegger H., Christ F. (1998). Attenuation of the near-infrared and red photoplethysmographic signal by different depth of tissues. Eur. J. Med. Res..

[9] Fitzpatrick T.B. (1988). The Validity and Practicality of Sun-Reactive Skin Types I Through VI. Arch. Dermatol..

[10] Guizar-Sicairos M., Thurman S.T., Fienup J.R. (2008). Efficient subpixel image registration algorithms. Opt. Lett..

[11] Van Gastel M., Stuijk S., de Haan G. (2016). New principle for measuring arterial blood oxygenation, enabling motion-robust remote monitoring. Sci. Rep..

[12] Moço A.V., Stuijk S., de Haan G. (2016). Skin inhomogeneity as a source of error in remote PPG-imaging. Biomed. Opt. Express.

[13] Fodor L., Ullmann Y., Elman M. (2011). Light Tissue Interactions.

[14] Kamshilin A.A., Nippolainen E., Sidorov I.S., Vasilev P.V., Erofeev N.P., Podolian N.P., Romashko R.V. (2015). A new look at the essence of the imaging photoplethysmography. Sci. Rep..

[15] Allen J. (2007). Photoplethysmography and its application in clinical. Physiol. Meas..

